# The role of alpha-rhythm states in perceptual learning: insights from experiments and computational models

**DOI:** 10.3389/fncom.2014.00036

**Published:** 2014-04-04

**Authors:** Rodrigo Sigala, Sebastian Haufe, Dipanjan Roy, Hubert R. Dinse, Petra Ritter

**Affiliations:** ^1^Department Neurology, Charité—University MedicineBerlin, Germany; ^2^Bernstein Focus State Dependencies of Learning, Bernstein Center for Computational NeuroscienceBerlin, Germany; ^3^Neural Plasticity Lab, Institute for Neuroinformatics, Ruhr-University BochumBochum, Germany; ^4^Minerva Research Group BrainModes, Max Planck Institute for Human Cognitive and Brain SciencesLeipzig, Germany; ^5^Berlin School of Mind and Brain, Mind and Brain Institute, Humboldt UniversityBerlin, Germany

**Keywords:** alpha rhythm, oscillations, attention, memory, learning, cognition, large-scale modeling

## Abstract

During the past two decades growing evidence indicates that brain oscillations in the alpha band (~10 Hz) not only reflect an “idle” state of cortical activity, but also take a more active role in the generation of complex cognitive functions. A recent study shows that more than 60% of the observed inter-subject variability in perceptual learning can be ascribed to ongoing alpha activity. This evidence indicates a significant role of alpha oscillations for perceptual learning and hence motivates to explore the potential underlying mechanisms. Hence, it is the purpose of this review to highlight existent evidence that ascribes intrinsic alpha oscillations a role in shaping our ability to learn. In the review, we disentangle the alpha rhythm into different neural signatures that control information processing within individual functional building blocks of perceptual learning. We further highlight computational studies that shed light on potential mechanisms regarding how alpha oscillations may modulate information transfer and connectivity changes relevant for learning. To enable testing of those model based hypotheses, we emphasize the need for multidisciplinary approaches combining assessment of behavior and multi-scale neuronal activity, active modulation of ongoing brain states and computational modeling to reveal the mathematical principles of the complex neuronal interactions. In particular we highlight the relevance of multi-scale modeling frameworks such as the one currently being developed by “The Virtual Brain” project.

## Introduction

Perceptual learning, a form of implicit learning and adult brain plasticity, allows us to tune our perception to efficiently select relevant sensory signals. It ultimately determines our success when adapting and interacting with the dynamic and complex environment. A glimpse into our daily life is enough to realize that learning efficacy varies greatly across human beings but also changes over time in a single person. A recent study on perceptual learning has shown that ongoing brain activity, more specifically, electrical oscillations in the alpha frequency band (~8–12 Hz), are able to predict up to 64% of the observed variability in the learning outcome in a perceptual task (Freyer et al., 2013). Although perceptual learning and brain function in general have been traditionally approached through the study of task-related brain activity, Freyer et al. (2013) and other recent studies demonstrate a growing awareness for a potential role of resting-state fluctuations (Biswal et al., 1995) for perceptual learning (Sigman et al., 2005; Lewis et al., 2009; Baldassarre et al., 2012; Freyer et al., 2013). Revealing the interaction between ongoing brain activity and learning could yield a new understanding of human cognition. This may lead to new strategies to modulate actively ongoing brain activity and to optimize brain states to manipulate the learning outcome in the clinical setting and everyday life.

In the present review we focus on the role that the alpha rhythm plays in perceptual learning. At the same time one can regard the alpha rhythm and perceptual learning as exemplary for other types of ongoing neural activity and cognition. They serve to illustrate how we can approach the endeavor to understand how the brain gives rise to cognition more generally. We show the need to build two bridges: One links neuronal activity to behavior and cognition; the other links neuronal activity to the underlying complex computational biophysical mechanisms spanning cellular, regional as well as large-scale network interactions. Imaging and neurofeedback studies addressed in this review are a good example of the first bridge. The computational modeling studies at different spatial scales presented here are examples of the second bridge.

Since cognition emerges through a temporal series of network operations, the temporal and the spatial aspects of brain activity need to be disentangled. Hence the alpha rhythm—like any other neural process of the brain—may play differential roles depending on the neuronal populations involved and the time point or temporal (cognitive) context. In other words, alpha oscillations may play different roles during the sequence of processes. They indicate or encode different aspects of information processing in the brain. In the present article we address how alpha oscillations may modulate perceptual learning. Based on recent computational models implementing biophysical plausible mechanisms, we hypothesize that high alpha ongoing activity in learning-related areas could play an “active” role, promoting learning by improving the encoding capabilities and memory formation. In more general terms, we illustrate the iterative process of empirical and modeling work that is necessary to infer knowledge about the interplay of intrinsic brain activity and external stimuli. This interplay constitutes the foundation of perceptual learning and in general of human cognition.

## Perceptual learning

Generally, learning occurs in different forms. Some forms explicitly require memorizing information (declarative learning, i.e., information we can describe), while others occur implicitly by exposition or practice (non-declarative or procedural learning, i.e., acquiring skills) (Gilbert et al., 2001). Perceptual learning is a form of implicit learning that can occur independent of declarative processes and is very similar in several aspects to non-declarative learning (Fahle and Poggio, 2002; Sagi, 2011). The term perceptual learning has a broad meaning. It captures learning at a great variety of conditions and occurs even in the absence of training (for a recent review see Beste and Dinse, 2013). When referring to perceptual learning in this review, we generally consider any change in perception and sensory guided behavior as a consequence of sensory experience (Fahle and Poggio, 2002).

**Figure 1:**
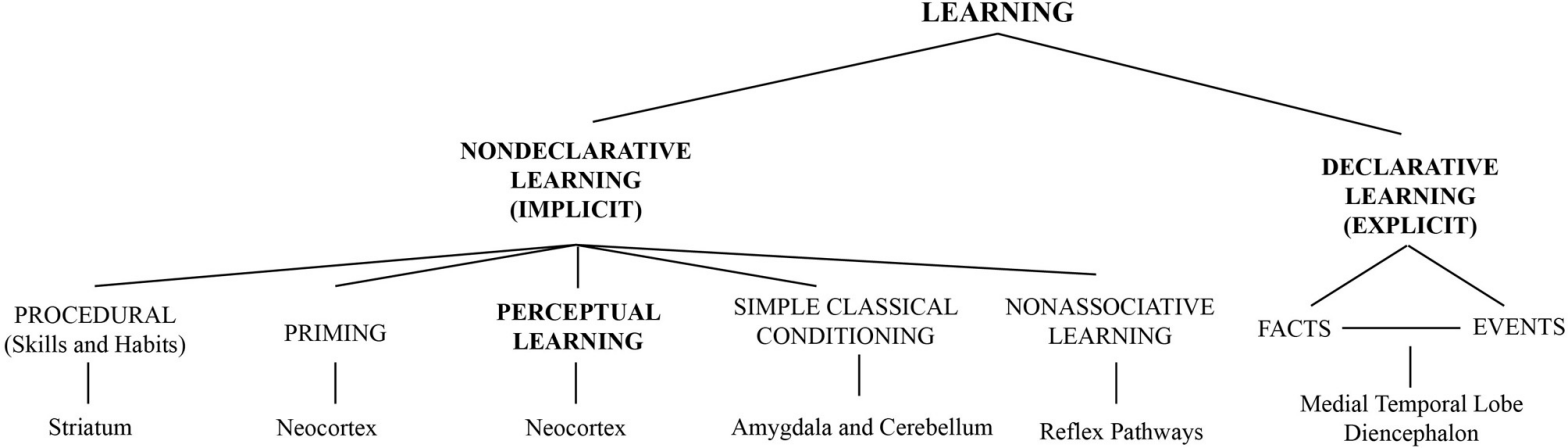
**Perceptual learning among other forms of learning classified according to the memory systems implicated (Figure adapted with permission from Squire and Zola, 1996. Copyright (1996) National Academy of Sciences, U.S.A)**. Brain regions traditionally associated with each form of learning/memory are presented at the bottom part of the figure.

In the visual system for example, learning can modify the perception of simple features such as orientation (Shiu and Pashler, 1992; Vogels and Orban, 1994), motion (Ball and Sekuler, 1982, 1987), contrast (Dorais and Sagi, 1997; Yu et al., 2004) as well as complex objects (forms) such as faces (Hussain et al., 2011, 2012; Herzog et al., 2012). A general scheme shown in Figure 1 illustrates how perceptual learning relates to other forms of learning. As different neuronal pathways are implicated in declarative and non-declarative forms of learning and memory (Squire and Zola, 1996), Figure 1 includes the main brain regions traditionally associated with these learning and memory processes (bottom part), locating perceptual learning in the neocortex. This association is important when considering regional interactions between ongoing alpha activity and perceptual learning.

### Cellular and regional substrates of perceptual learning

Perceptual learning can restructure cortical networks on a large-scale accessible by non-invasive imaging methods within hours or even minutes (e.g., Caroni et al., 2012; Freyer et al., 2012a; Sagi et al., 2012), and can result in functional changes throughout the cortex (Gilbert et al., 2009; Sasaki et al., 2010). Plastic changes involved in improving for example the discrimination of an attribute are likely to occur at the primary sensory processing level representing specific features, i.e., in cortical regions where receptive fields are selective to those attributes (Tsodyks and Gilbert, 2004; Carmel and Carrasco, 2008). Nevertheless, alterations induced by perceptual learning can go beyond primary and secondary sensory cortices. In the visual system for example, numerous regions have been implicated in visual perceptual learning such as V1/V2, V3, V4, the middle temporal area (MT), the lateral intraparietal area (LIP), and areas related to attention, decision making, and default mode networks (Yang and Maunsell, 2004; Mukai et al., 2007; Law and Gold, 2008; Lewis et al., 2009; Sasaki et al., 2010; Shibata et al., 2011). Plastic changes induced by perceptual learning may occur widespread throughout the cortex or only in local neuronal dynamics (Jones et al., 2007; Sasaki et al., 2010).

Perceptual learning is considered to be a manifestation of external stimulus-driven neural plasticity in the brain (Carmel and Carrasco, 2008) and has recently been linked to long-term potentiation (LTP) and long-term depression (LTD) (e.g., Beste et al., 2011; Sale et al., 2011; Aberg and Herzog, 2012; Ditye et al., 2013). LTP and LTD are long-lasting alterations in the efficiency of synaptic transmission, typically induced by brief periods of coordinated, high (LTP), or low (LTD) frequent neuronal activity at a synapse (see Malenka and Bear, 2004 for a review). Since the pioneering work of Bliss and colleagues (1973), LTP and later LTD became key candidates for neuronal plasticity and learning in almost any part of the mammalian brain (Bliss and Gardner-Medwin, 1973; Bliss and Lomo, 1973; Nicoll et al., 1988; Lynch, 2004; Malenka and Bear, 2004; Citri and Malenka, 2008).

### Role of attention in perceptual learning

One candidate for a link between alpha and perceptual learning is attention (see section Alpha Rhythm and Attention). Generally, learning, attention, and memory appear to be strongly related (Gilbert et al., 2001). More than 30 years ago, Schneider and colleagues (Schneider and Shiffrin, 1977; Shiffrin and Schneider, 1977) proposed that learning the automatic detection of visual categories reduces the dependence of performance from attentional control, resulting in the automatization of the task. Later on, several studies in the nineties reported that attention is often required for the consolidation of non-declarative memory in visual perceptual learning (Shiu and Pashler, 1992; Ahissar and Hochstein, 1993; Fahle and Morgan, 1996; Braun, 1998; Ito et al., 1998). More recent studies observed visual perceptual learning exclusively when subjects were consciously involved in a task, suggesting the interplay of top-down guided processes (Ahissar and Hochstein, 2004) with relevant influence of attention (Roelfsema et al., 2010). In experiments in which subjects were asked to give a response depending on different visual features of the presented stimuli, it was demonstrated that observers show perceptual learning only for the features that were attended (Shiu and Pashler, 1992; Ahissar and Hochstein, 1993). In this line, long-range coupling has been reported between frontal and sensory areas during attention (Gregoriou et al., 2009). At the same time, the alpha rhythm has been implicated in the long-range communication between cortical areas (Von Stein and Sarnthein, 2000). As detailed further below, electrophysiological studies in cats investigating the coupling between different brain areas identified signals in the theta-alpha range (4–12 Hz) to be relevant in top-down modulation of incoming stimuli (Von Stein et al., 2000). However, evidence is accumulating indicating that perceptual learning can also occur when subjects are not involved in a task and exert no conscious effort. In these cases top-down attention has less or no influence (Zajonc, 1968; Skrandies and Fahle, 1994; Watanabe et al., 2001, 2002; Seitz and Watanabe, 2003; Nishina et al., 2007; Gutnisky et al., 2009; Seitz et al., 2009; Rosenthal and Humphreys, 2010; Shibata et al., 2011). In some cases this type of perceptual learning, usually referred to as “task-irrelevant perceptual learning,” requires reward and reinforcement signals (Seitz et al., 2009 for example used, food and water deprivation to manipulate the reward) to consolidate information about incoming stimuli (see Sasaki et al., 2010 for review).

### Perceptual learning via time-dependent sensory stimulation

Recent work suggests that active training may not be required in perceptual learning (see Beste and Dinse, 2013 for review). Instead, changes in perception can be effectively induced by mere exposure to repetitive sensory stimulation (RSS). Such training-independent sensory stimulation induces lasting changes in perception and goal-directed behavior without any explicit task training. RSS protocols are regarded as “passive stimulation” since no attentional effort is required (Dinse et al., 2011). Ragert et al. (2008) translated stimulation protocols used in brain slice preparations into tactile high-frequency stimulation (HFS) to drive perceptual changes. HFS consisted of pulse trains that were applied to the tip of the right index finger with a stimulation frequency of 20 Hz using either cutaneous or electrical stimulation. Ragert et al. (2008) found that 20 min of HFS induced a lowering of tactile discrimination thresholds, indicating improved tactile acuity, whereas the left index finger of the non-stimulated hand showed no changes in acuity.

**Figure 2:**
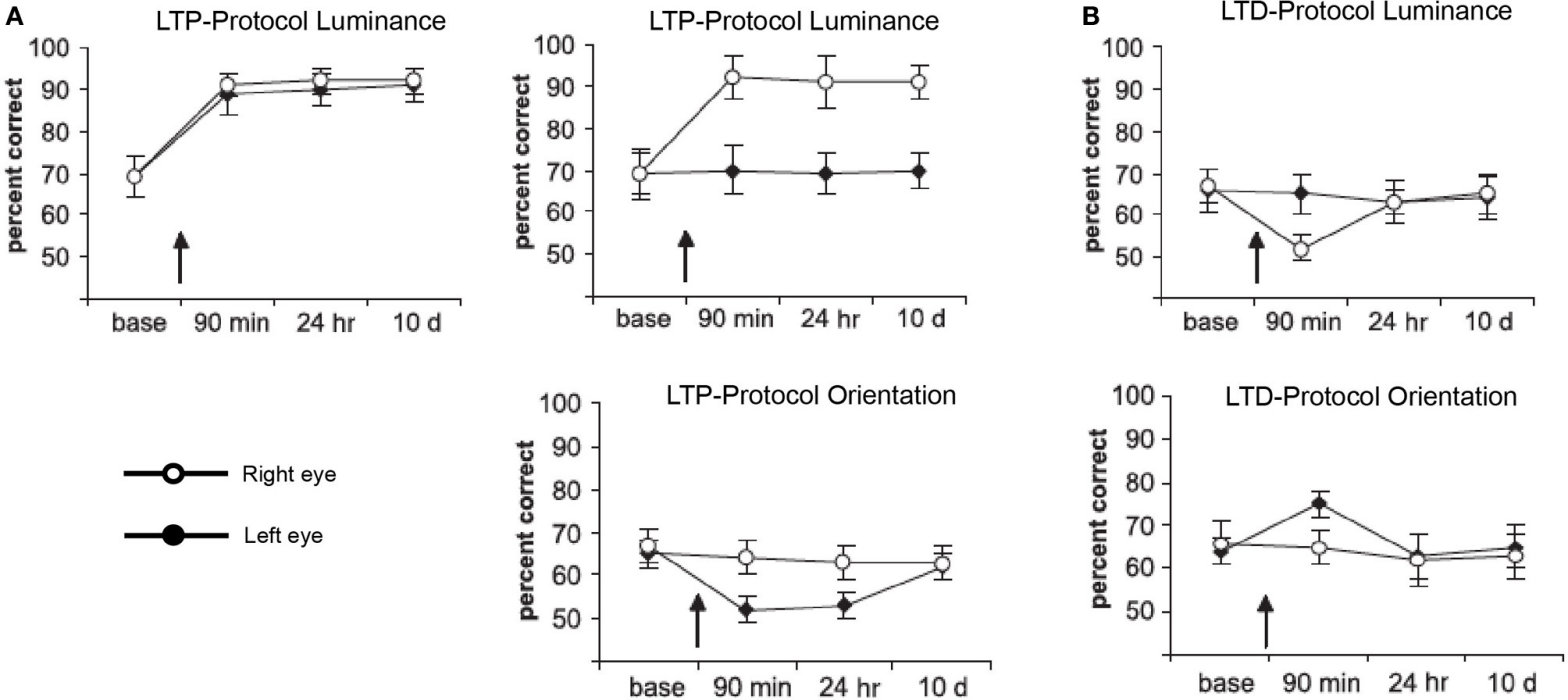
**Performance in a luminance detection task in which changes in the orientation of the stimuli (bars) were used as a distractor in some so-called “competitive trials.”** Performance in competitive trials is plotted for each time point: baseline (base), 90 min later (90 min), 24 h later (24 h), and 10 days later (10 d). Each panel shows the results of different experiments depending on the visual-stimulation protocols used. Stimulation of the relevant (luminance) or irrelevant (orientation) feature could be LTP **(A)**- or LTD **(B)**-like, applied to both (bilateral) or only the right eye (unilateral). Black and white circles represent performance on the right and left side of the fixation cross, respectively. Error bars are standard errors of the mean. Figure adapted with permission from Beste et al. (2011).

For the visual modality, recent studies have shown that time-dependent stimulation can affect visual performance (Beste et al., 2011; McMahon and Leopold, 2012). Beste et al. (2011) used an LTP- and LTD-like visual stimulation to improve or impair performance of a change-detection task (see Figure 2). Task relevant or irrelevant features of the stimuli were used for high- or low-frequency stimulation. HFS (20 Hz) comprising a 40 min presentation of the relevant feature stimuli caused an increase in performance (Figure 2A). Low-frequency stimulation (LFS, 1 Hz) involving the relevant feature, as well as HFS using the irrelevant feature caused impairment (Figure 2B). In another approach, time-dependent stimulation was applied in human observers to mimic spike-timing-dependent plasticity to induce plasticity in high-level vision (McMahon and Leopold, 2012). The authors used asynchronous presentation of faces to influence the perception of face identity.

### EEG and bold correlates of perceptual learning

The neural correlates of perceptual learning in humans have been investigated in several studies using functional magnetic resonance imaging (fMRI) or electroencephalography (EEG) recordings. The majority of studies have focused on the visual modality (see Sagi, 2011 for a review). Using a paradigm in which subjects were trained to discriminate different visual textures, it was shown that fMRI BOLD (blood-oxygen-level dependent) responses elicited by the trained textures in V1 were stronger when viewed with the trained eyed as compared to the untrained eye (Schwartz et al., 2002). Changes in V1 as a consequence of training were also confirmed by subsequent studies (e.g., Furmanski et al., 2004; Walker et al., 2005; Yotsumoto et al., 2009). By comparing subjects' ability to learn image-statistical regularities and distinguish targets in clutter, another study identified the BOLD correlates of two different brain plastic signatures that underlie these two forms of visual perceptual learning (Zhang and Kourtzi, 2010). In addition to fMRI studies, plasticity in low visual areas as a consequence of perceptual training has also been investigated using other methods such as EEG (e.g., Skrandies and Fahle, 1994; Casco et al., 2004; Furmanski et al., 2004; Walker et al., 2005; Pourtois et al., 2008) and diffusion tensor imaging (DTI) (Yotsumoto et al., 2010). In a recent study, Mayhew et al. (2012) compared human performance with the performance of pattern classifiers using fMRI/EEG signals recorded simultaneously. They found evidence of distinct brain mechanisms that underlie the improvement of the ability to perceive uncertain (i.e., noisy) visual stimuli.

The neural correlates of perceptual changes induced through RSS protocols have also been found in the somatosensory cortex (Pleger et al., 2001; Freyer et al., 2013). Assessing the effect of a RSS protocol on tactile discrimination behavior and somatosensory-evoked potentials, Pleger et al. (2001) demonstrated a correlation between individual perceptual improvement and localized activity in somatosensory cortex. These effects were also confirmed using fMRI where cortical reorganization in primary and secondary somatosensory cortex (S1 and S2) was observed after the same stimulation protocol (Pleger et al., 2003).

### Computational models of perceptual learning

The majority of computational models that describe neuronal interactions within and between populations underlying perceptual learning focus primarily on the visual system. These models can be classified into two groups. The first group of models implements learning in early visual processing areas, such as V1, inspired by the retinotopic organization of those areas (e.g., Adini et al., 2002; Teich and Qian, 2003; Zhaoping et al., 2003). The second group of models achieves learning by changing the representation of higher-level cortical areas or by modifying the connections between low-level areas and higher-level association areas, such as those implicated in decision-making processes (Poggio et al., 1992; Dosher and Lu, 1998; Sigala et al., 2005; Serre et al., 2007; Lu et al., 2010).

The wide range of modeling approaches raises a major open question: does perceptual learning induce changes in early sensory areas or rather a reweighting of connections between primary sensory cortices and higher-cortical areas involved in decision-making processes. Most of the computational models on perceptual learning use feedforward models with recurrent interactions (Poggio et al., 1992; Dosher and Lu, 1999; Eckstein et al., 2004; Sigala et al., 2005; Serre et al., 2007). In such architectures learning can be implemented using a training signal that in principle guides the reweighting of the connections between units at different processing stages. In these models, feedback about decision errors is fundamental (Poggio et al., 1992; Herzog and Fahle, 1997, 1999). Other learning strategies have followed an unsupervised or semi-unsupervised approach. A possibility in such cases is to build feature detectors capable of changing their tuning properties, and to adapt them to the statistical properties of the training set (Sigala et al., 2005; Serre et al., 2007). In a recent publication, Solgi et al. (2013) propose a model that explains generalization (transfer) learning effects to untrained features. According to this model, transfer learning occurs since particular tasks are able to trigger neuronal recruitment in lower-feature and higher-association areas, relevant for both the trained and the untrained conditions.

## The alpha rhythm and its impact on information processing

In this section we focus on different aspects of the alpha rhythm that may be relevant for the understanding of its role in perceptual learning. We address the cellular and regional correlates of alpha oscillations. We highlight generative computational models that yield alpha activity and explore accumulated evidence linking alpha oscillations to cognition, generally agreeing with the available hypothesis that situate the alpha rhythm as an inhibitory mechanism which gates resources necessary for information processing (Jensen and Mazaheri, 2010).

### General aspects of the alpha rhythm

The alpha rhythm refers to brain oscillations within a frequency range of 8–12 Hz. This rhythm was first observed when Hans Berger recorded electrical activity from the scalp (EEG) in 1929 (Berger, 1929). Other frequency bands discovered later were also labeled using Greek letters, the boundaries of which were arbitrarily drawn: delta, 0.5–4 Hz; theta, 4–8 Hz; beta, 12–30 Hz; gamma, >30 Hz (Buzsaki, 2006). Opening and closing the eyes modulates the amplitude of alpha oscillations (see Pfurtscheller et al., 1996 for a review). Given the observed attenuation (also referred to as “desynchronization”) of the alpha band signal caused by opening the eyes, some investigators concluded that the alpha band reflects an “idling” state in which the underlying cortical regions are not engaged in any task or processing of sensory information (Pfurtscheller et al., 1996). Nowadays the “idling” role of alpha oscillations has been overtaken by the so-called inhibition hypothesis (see Klimesch et al., 2007 for a review). This hypothesis is supported by the observation that the amplitude of alpha oscillations is suppressed in specialized sensory areas when devoted to the processing of sensory stimuli (Nikouline et al., 2000) while it emerges in areas that are not explicitly involved in the respective task (Worden et al., 2000; Kelly et al., 2006; Thut et al., 2006).

Although alpha oscillations are most prominent in visual areas, i.e., they exhibit highest amplitudes in electrodes placed over occipital brain areas, they are generally widespread in the cortex but regionally attenuated depending on different stimuli and tasks (Buzsaki, 2006, p. 198–200). Hence alpha rhythms have presumably distinct functional roles and mechanisms of generation. Alpha waves can be recorded in electrodes near the frontal eye fields, cortical areas responsible for eye movements (Niedermeyer and Da Silva, 2004, Ch. 9), above the sensory-motor cortical area (usually referred to as μ, “Rolandic” or somatosensory alpha rhythm) (Gastaut, 1952; Kuhlman, 1978; Salmelin and Hari, 1994), over the supplementary motor area (Pfurtscheller and Berghold, 1989), as well as above the auditory (midtemporal) cortex (“tau” rhythm) (Lehtela et al., 1997). In view of these findings it is very likely that synchronized oscillations in the alpha band are a common feature of cortical activity especially in sensory cortices, making them key candidates for modulating cognitive functions such as perceptual learning.

### Cellular and regional substrates of the alpha rhythm

Initial evidence suggested that alpha oscillations originate solely from thalamo-cortical interactions (Andersen and Andersson, 1968). More recently, Bollimunta et al. (2011) argued that alpha activity in V1 appears to be generated by thalamo-cortical interactions that possibly also influence alpha oscillations in higher cortical areas along the stream of visual processing. Neurons in the thalamus possess the biophysical features (Lopes Da Silva et al., 1980; Hughes and Crunelli, 2005; Lorincz et al., 2009; Bollimunta et al., 2011; Hughes et al., 2011) and the anatomic connectivity (Jones, 2002) that enable them to shape cortical alpha oscillations. In addition to the talamic lateral geniculate nucleus (LGN), which is supposed to drive occipital alpha rhythms (Hughes and Crunelli, 2005), especially the pulvinar nucleus is considered to exert an influence over cortical alpha rhythms (Lopes Da Silva et al., 1980) modulating the synchrony between cortical areas according to the locus of attention (Saalmann et al., 2012).

Other studies locate the origin of alpha oscillations in deep layer cortical neurons and networks (Da Silva et al., 1973; Lopes Da Silva and Storm Van Leeuwen, 1977; Steriade et al., 1990; Flint and Connors, 1996; Castro-Alamancos and Rigas, 2002; Bollimunta et al., 2011; Ronnqvist et al., 2013). An *in vitro* preparation by Silva et al. (1991) showed that synchronized oscillations especially in the alpha band can be generated solely by neurons of cortical layer 5, which possess all the necessary intrinsic properties and synaptic connections to generate alpha oscillations. Bollimunta et al. (2008) found alpha generators in layers 3, 4, and 5 of the macaque visual cortex and suggested that in general layers with higher spontaneous activities seem to contain the pacemakers of the alpha rhythm. The sites of alpha generators differ not only along the stream of processing but also for different modalities. In the primary motor cortex (M1) for example, oscillatory activity in the alpha range (Rolandic μ rhythm) is supposedly mainly generated in layer 3 (Ronnqvist et al., 2013).

Numerous feedforward and feedback modules (Callaway, 1998; Jiang et al., 2013) enable complex interactions between cortical layers and columns. Therefore, cortical oscillations in different frequency bands are closely linked. A recent study by Spaak et al. (2012) for example, showed an “intimate relationship” between alpha and gamma band dynamics within the primate V1 cortical microcircuits. Driven by deep layer alpha generators, gamma band activity in superficial granular and supragranular layers is modulated in a suppressive, phase-specific manner (Spaak et al., 2012).

**Figure 3:**
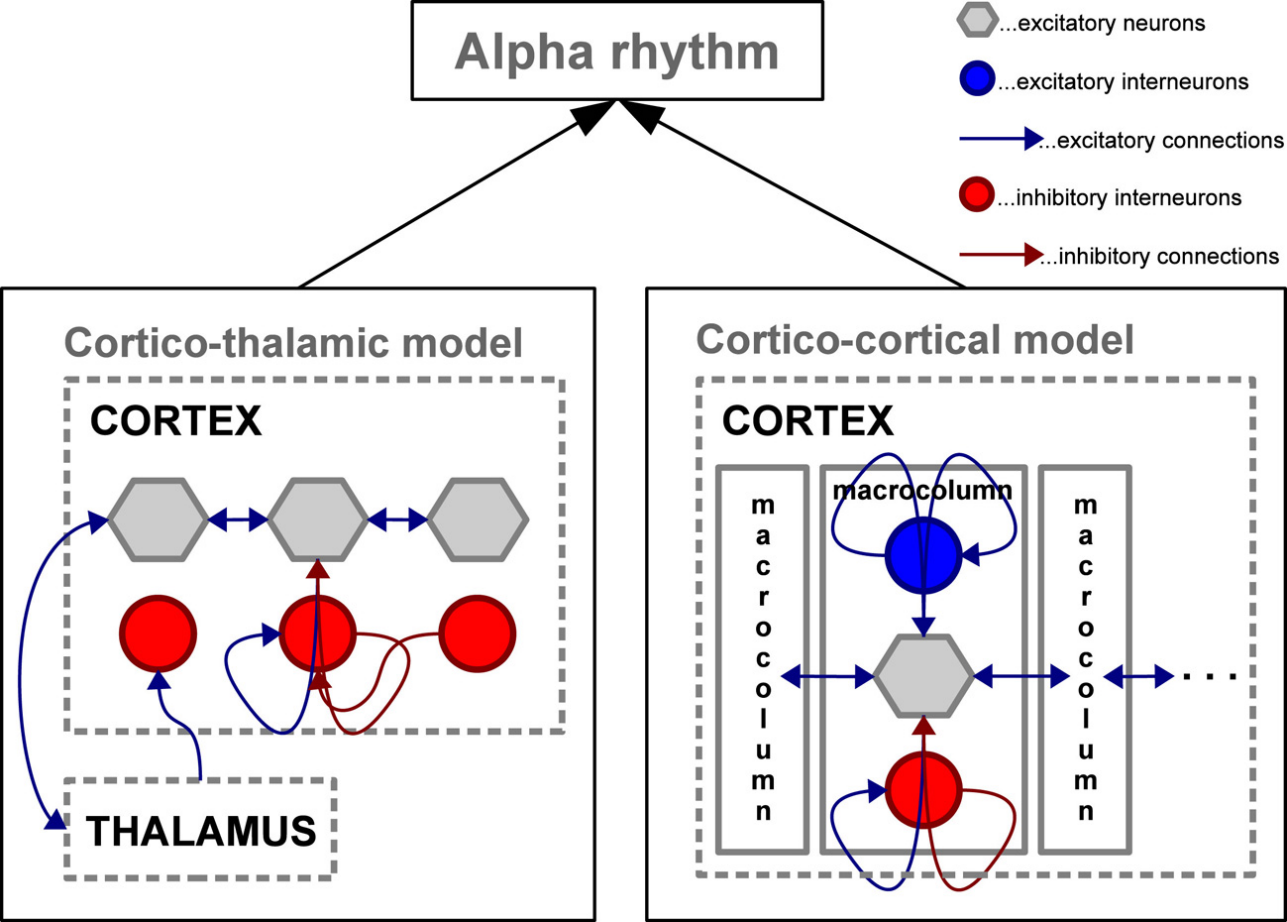
**Schematic depiction of two mesoscopic computational models capable of simulating oscillations in the alpha frequency range**. Left: Cortico-thalamic model adapted from Freyer et al. (2011) involving the thalamus to generate alpha activity in a network of cortical excitatory neurons and inhibitory interneurons; Right: Cortico-cortical model adapted from Naruse et al. (2010) generating alpha activity without the thalamic control by interconnecting cortical macro-columns composed of excitatory pyramidal neurons accompanied by excitatory and inhibitory interneuron networks.

Taken together, empirical evidence demonstrates that the alpha rhythm can be generated through cortical interactions with or without the need for thalamic input. Results vary substantially depending on whether neural assembles are studied *in vitro* or in the intact brain, as well as on the particular animal model, task and hence brain region investigated. As will be shown in the section on microscopic, mesoscopic, large-scale, and full-brain computational models, alpha activity can be generated with or without the need for thalamic activity, i.e., using exclusively cortical interactions (see Figure 3).

### Imaging the alpha rhythm and other features of ongoing brain activity

**Figure 4:**
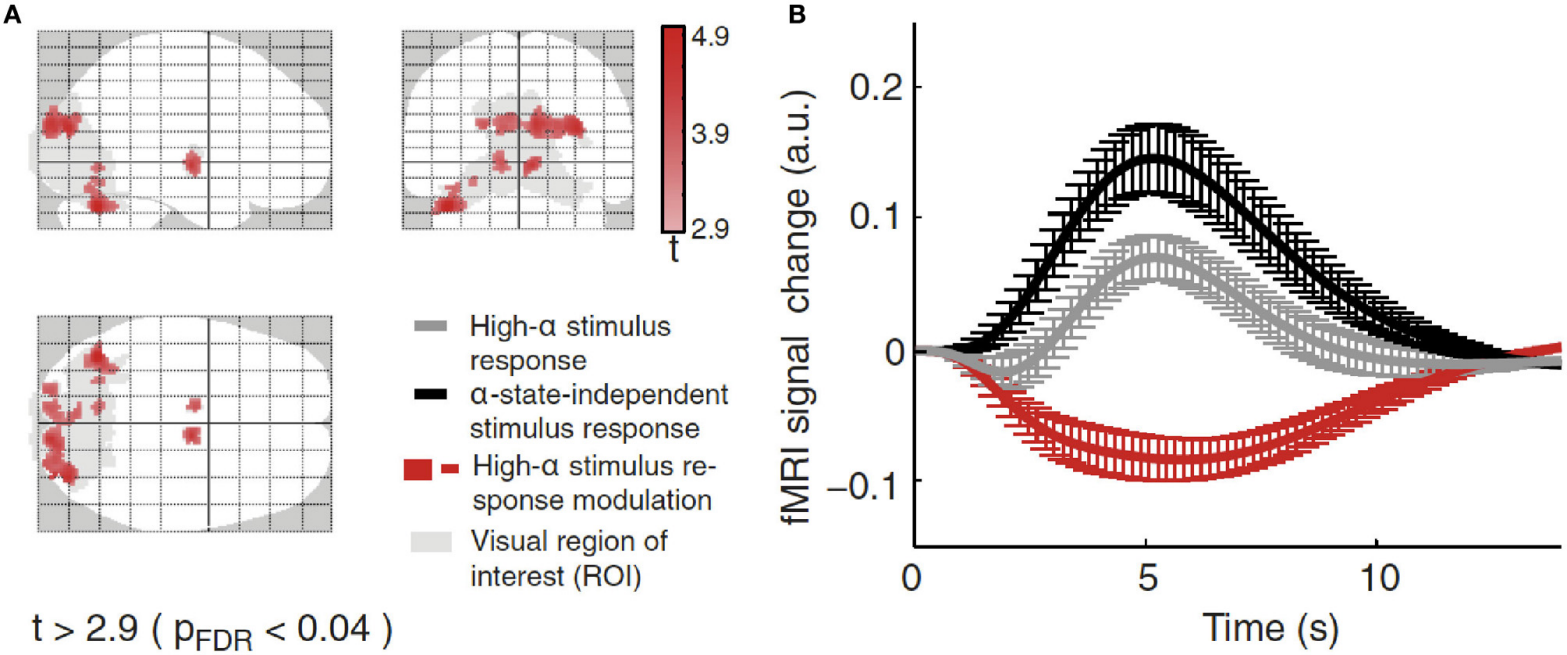
**(A)** BOLD deactivations (red) within a visual ROI (gray) projected onto a brain template. **(B)** Time courses of the responses in the clusters selected showing responses in the high alpha-state (black line), alpha-independent (gray line) conditions, and the difference of both (high-alpha stimulus response modulation, red line). Figure adapted with permission from Becker et al. (2011).

Using combined recordings of EEG and BOLD fMRI activity (Ritter and Villringer, 2006; Becker et al., 2009; Ritter et al., 2010) it has been possible to observe thalamic and cortical BOLD activity in relation to the alpha rhythm in human subjects. Several studies have reported higher alpha-rhythm amplitudes in occipital (Goldman et al., 2002; Moosmann et al., 2003; Feige et al., 2005; Goncalves et al., 2006; De Munck et al., 2007; Difrancesco, 2008) and sensorimotor cortex (Ritter et al., 2009) associated with negative BOLD fMRI signals in sensory areas (Ritter and Villringer, 2002). There exist distinct relations between fMRI resting-state network (RSN) fluctuations and EEG global fields (i.e., average activity of all EEG channels) for different frequency bands (Mantini et al., 2007). Considering also the space structure of the EEG, i.e., identifying ICA components with distinct topographic distributions, reveals that alpha oscillations of a single frequency band yet with independent time structure and different space structure (topography) may be linked to different BOLD-RSNs (Becker et al., 2009). This may explain topographic and qualitative variability of fMRI correlates of EEG rhythms. De Munck et al. (2007) have demonstrated such variability. Other groups reported alpha correlates in fronto-parietal networks (Laufs et al., 2003a,b, 2006) or over Rolandic (sensorimotor) areas (Ritter and Becker, 2009). Simultaneously recording BOLD and EEG signals, Scheeringa et al. (2011) observed that the BOLD response elicited by a short visual stimulus was modulated by the phase of the ongoing alpha oscillations. Additionally for evoked potentials, alpha amplitude (Becker et al., 2008; Reinacher et al., 2009) and phase dependencies have been demonstrated (but see Ritter and Becker, 2009). Using EEG-triggered sensory stimulation (Reinacher et al., 2009) together with simultaneous BOLD measurements, another study demonstrated that spontaneous alpha-rhythm fluctuations in power could largely explain the evoked fMRI response variance observed in extrastriate, thalamic, and cerebellar areas (Becker et al., 2011). As depicted in Figure 4, Becker et al. (2011) showed that BOLD responses to visual stimuli in clusters of visual responsive voxels are modulated by the state of the ongoing alpha activity. Technical advances in simultaneous EEG–fMRI acquisition nowadays allow recording of a wide range of oscillations including the gamma band and subtle ultrafast population spikes (Ritter et al., 2008; Freyer et al., 2009b) and setting those different frequency bands in relations in terms of their spatial and temporal features (Schultze-Kraft et al., 2011).

### Alpha rhythm and perception

In the last decade several studies investigating the functional role of alpha oscillations have focused on their relation with perception (summary in Table 1). Using visual stimuli near the detection threshold, Ergenoglu et al. (2004) observed that trials with detected stimuli contained significantly less power in the alpha band than trials with undetected stimuli. Hanslmayr et al. (2007) also showed that successful perceptual performance in a visual task is related to little alpha power during the prestimulus interval. Investigating the effect of competing stimuli in the somatosensory modality, Schubert et al. (2009) demonstrated that some features of the ongoing EEG activity (e.g., ~10 Hz) before stimulus presentation predicted whether weak stimuli could be consciously perceived after masking it with a stronger distractor.

**Table 1:** **Overview of studies showing correlations between features of alpha oscillations (i.e., amplitude, power, phase) and perception**.

	**Alpha and perception**				
	**Modality/task**	**Region**	**Time interval**	**Correlation with behavioral performance**	
				**Power or amplitude**	**Phase**
Ergenoglu et al., 2004	Visual/detection	Parietal/occipital	Stimulation	Negative	
Hanslmayr et al., 2007	Visual/discrimination	Parietal/occipital	Pre-stimulus	Negative	✓
Schubert et al., 2009	Somatosensory/detection (masking)	Pericentral sensorimotor	Pre-stimulus	Negative	
Babiloni et al., 2006	Visual/visuospatial	Frontal, parietal and occipital	Pre-stimulus	Positive	
Linkenkaer-Hansen et al., 2004	Somatosensory/detection	Sensorimotor parietal	Pre-stimulus	Intermediate amplitude. Large amp.	
Van Dijk et al., 2008	Visual/discrimination	Parietal/occipital	Pre-stimulus	Increase leads to less sensitivity	
Lange et al., 2013	Visual + somatosensory/discrimination	Occipital	Pre-stimulus	Negative	
Callaway and Yeager, 1960	Visual/reaction-time	Occipital	Stimulation		✓
Dustman and Beck, 1965	Visual/reaction-time	Occipital	Stimulation		✓
Mathewson et al., 2009	Visual/detection	Posterior	Stimulation	Positive	✓
Palva et al., 2005	Somatosensory/detection	Somatosensory			✓
Becker et al., 2011	Visual/detection	Posterior	Pre-stimulus change	Positive	

*For each study (rows) we indicate (columns) the sensory modality investigated, task employed, brain region implicated, and time interval analyzed. Additionally we indicate whether the power or amplitude of the alpha band positively (+) or negatively (−) correlated with behavioral performance. In the last column on the right part we used a tick to indicate that performance correlated with alpha phase*.

Instead of lower alpha activity, Babiloni et al. (2006) observed a stronger power component in frontal, parietal, and occipital alpha in trials in which stimuli were perceived (interestingly Linkenkaer-Hansen et al., 2004 found an inverted U-shape association between alpha power and consious detection in the somatosensory modality). Using magnetoencephalograms (MEG) and analyzing signals at the source level using spatial filters, Van Dijk et al. (2008) showed in a visual discrimination task that an increase in posterior alpha power previous to stimulus presentation correlated with less sensitivity. In a paradigm in which somatosensory stimulation was used to bias visual perception, Lange et al. (2013) found that prestimulus alpha activity is related to improved perception of illusory stimuli. The authors suggested that alpha activity is generally linked to enhancement of excitability of visual cortex, rather than improving perception as such.

While all studies mentioned above concentrated on alpha amplitude, other studies have focused on studying the phase of alpha signals (Callaway and Yeager, 1960; Dustman and Beck, 1965; Varela et al., 1981; Becker et al., 2008; Busch et al., 2009; Mathewson et al., 2009; Ritter and Becker, 2009; Busch and Vanrullen, 2010; Hanslmayr et al., 2011). Mathewson et al. (2009) demonstrated that flashed stimuli were more likely to be detected when presented at the positive peak than at the negative peak of the alpha waves in trials where alpha amplitude was high. Recent studies further highlighted the importance of ongoing oscillatory alpha phase in the perception of illusory (Dugue et al., 2011) and near-threshold visual stimuli (Mathewson et al., 2011; Vanrullen et al., 2011), as well as in the conscious access to visual stimuli (Pincham and Szucs, 2012). Using near-threshold tactile stimuli, Palva et al. (2005) showed stimulus locking in the alpha band (8–14 Hz) in somatosensory regions, dominant for consciously perceived stimuli but almost unobservable for unperceived stimuli.

Besides their role in target detection, alpha oscillations have been also found to correlate with multi-stable perception. A decrease of alpha power has been observed to precede perceptual reversals of bistable visual stimuli, such as the Necker cube (Isoglu-Alkac et al., 2000; Isoglu-Alkac and Struber, 2006). Using ambiguous motion, Mathes et al. (2010) reported a perceptual reversal-related desynchronization of alpha activity in posterior locations. This is interesting since it highlights the potential relevance of alpha activity for intrinsic brain state switches (see Freyer et al., 2009a,b for a characterization of alpha modes and computational models that capture those). Those alpha state switches may occur unrelated to external events but have significant input on our perception and cognition.

### Alpha rhythm and attention

Using audiovisual stimuli, (Foxe et al., 1998) observed that the alpha rhythm is related to visual attentional gating in the presence of a relevant auditory stimulus. In a spatial cueing paradigm with purely visual stimuli, Worden et al. (2000) noticed that alpha activity during the cue-stimulus interval increased in the occipital cortex contralateral to the “to-be-ignored” direction (ipsilateral to the cued location). This pattern of results has been interpreted as a signature of an inhibitory process that helps to prepare activity in places where stimuli are expected and visual processing is required (Worden et al., 2000; Kelly et al., 2006; Handel et al., 2011).

Other investigators have found a decrease in alpha activity (event related desynchronization or ERD) over posterior electrodes contralateral to the attended side (Kelly et al., 2006; Thut et al., 2006; Rihs et al., 2007; Wyart and Tallon-Baudry, 2008; Yamagishi et al., 2008; Mathewson et al., 2009; O'connell et al., 2009; Rihs et al., 2009; Snyder and Foxe, 2010; Mo et al., 2011). Since this desynchronization effect correlated with subsequent behavioral performance, alpha ERD has been associated with an enhanced excitability of cortical areas in charge of processing stimuli in the attended visual field. Supporting this idea, Rohenkohl and Nobre (2011) have also reported alpha ERD in a task in which temporal expectations were manipulated.

Idling states of alpha are also investigated in terms of directed and non-directed attention (non-specific alertness). To explain the role of alpha in non-directed attention Sadaghiani et al. (2010) proposed a generalized “windshield wiper” mechanism. The authors suggest that alpha oscillations rhythmically and synchronously clear sensory information on a rapid time-scale from specific channels that are require for the detection of novel and relevant incoming sensory information (Sadaghiani et al., 2010). If the above hypothesis is indeed true, then this would suggest that alpha activity can bias cortical processing in favor of strong and recent sensory signals. In both cases, non-directed and directed attention, alpha increases responsiveness of some areas but decreases responsiveness of others. Low-frequency but high amplitude alpha oscillations show larger impact on target populations. Yet during desynchronization of faster oscillations (such as gamma) population gain increases, most likely, in accord with the gradual release of inhibition and amounts to specific and focal disruption of this global effect. The abovementioned theory for selective attention is supported by a large pool of literature showing that in directed attention, regions representing the attended site exhibit ERD while the others (non-attended) exhibit ERS (Klimesch, 2012).

### Causal role of the alpha rhythm

Alpha activity correlates with important processes underlying information processing, including perceptual learning. Yet, whether these correlations indicate a causal relation between behavior and the alpha rhythm remains unknown. Intervening brain activity through neurofeeedback and/or non-invasive brain stimulation can shed light on the causal relation between alpha rhythm and perceptual learning. Transcranial magnetic stimulation (TMS) has proven to successfully modulate ongoing alpha oscillations, eventually modulating visual perception (Romei et al., 2010; Thut et al., 2011; see Neuling et al., 2012 for the auditory modality; Romei et al., 2012; see Thut et al., 2012 for a review). Neurofeedback training on the other hand, is a protocol in which subjects learn to generate specific brain patterns of activity interpreted through a so-called “brain-computer interface” (BCI). Neurofeedback has been used in clinical applications (e.g., Hardt, 1978; Saxby and Peniston, 1995; Birbaumer et al., 1999; Gruzelier et al., 1999; Sterman, 2000) but also to boost the performance of healthy subjects in a wide variety of tasks (see Vernon, 2005 for a review) such as those reflecting cognitive performance, for example working memory (Vernon et al., 2003) and mental rotation tasks (Vernon et al., 2003; Hanslmayr et al., 2005; Zoefel et al., 2011). Ros et al. (2010) combined neurofeedback training and TMS to show that alpha oscillations contribute significantly to cortical plasticity in motor cortex, causing brain changes that outlast their phase of entrainment. The authors speculate that the plasticity effects they observe could be explained by mechanisms related to long-term and short-term potentiation, which in turn could interact with alpha oscillations in the context of perceptual learning.

A number of reasons have been proposed to explain the difficulty when using neurofeedback to control the alpha rhythm and cognitive performance. One is the fact that the peak of the alpha frequency varies among subjects, the identification of which is necessary to select the exact frequency band that needs to be enhanced (Klimesch et al., 1993). The alpha frequency can be further separated into different sub-bands of differential relevance for different cognitive tasks. These sub-bands include lower alpha 1 (6–8 Hz), medium alpha 2 (8–10 Hz), and upper alpha (10–12 Hz). Lower alpha is related to attentional demands, whilst the upper alpha is associated with semantic memory (Klimesch et al., 1994, 1998). By making such a distinction between alpha sub-bands it has been recently shown, for example, that training of the upper alpha band increases cognitive control in a mental rotation task (Zoefel et al., 2011).

The computer-model guided self-regulation of precisely localized brain activity with control of high-resolution temporal information appears to be a promising approach to controlling cognitive performance. Combining EEG and fMRI utilizing their synergies in terms of spatial and temporal resolutions with analytical tools that account for the space-time structure of the brain (Schultze-Kraft et al., 2011) seems particularly appealing in this context. The development of real-time fMRI (rtfMRI) techniques (Decharms, 2008; Laconte, 2011; Weiskopf, 2012) and real time EEG during fMRI (Becker et al., 2009) makes it feasible in principle. The online self-regulation of brain areas localized through fMRI and EEG can significantly contribute to the understanding of the causal relations between physiology and behavior.

### Computational models of the alpha rhythm

Computational models of brain function exist with different granularity depending on the targeted neural processes. In this section we highlight a selection of microscopic, large-scale, and full-brain computational models of the alpha rhythm.

#### Microscopic neuronal network models

Microscopic models of the alpha rhythm deal with important cellular processes leading to changes in synaptic activity. Fundamental biophysical insights gained from *in vitro* experiments (Silva et al., 1991; Flint and Connors, 1996) have been summarized in models of neocortical networks of excitatory and inhibitory neurons that display remarkable concordance with alpha-like rhythms (Jones et al., 2000; Karameh et al., 2006; Neymotin et al., 2011). In a model proposed by Jones et al. (2000), inward currents (known as h and T currents, modulating the period in which neurons membrane potential remains in a subthreshold state) in layer 5 pyramidal neurons were able to regulate the alpha rhythm and exhibit asynchronous firing patterns that matched the experimentally observed spatial asynchrony of the alpha rhythm. Karameh et al. (2006) in their model showed that modifications of intrinsic currents of layer 5 cells led to resonance-like behavior in neuronal populations. More recently Vijayan and Kopell (2012) proposed a 2-fold model of thalamic alpha activity governing cortical alpha to either facilitate processing or prevent stimuli from reaching the cortex. Interestingly, Vijayan et al. simulated these thalamic processes by mimicking the action of muscarinic acetylcholine receptor or metabotropic glutamate receptor 1 agonists on thalamic reticular, thalamocortical, and high-threshold thalamocortical cells.

#### Mesoscopic and large-scale network models

Modeling attempts using large-scale networks to understand the emergence of cognitive states rely heavily on the approximation of the dynamics of a neural ensemble. In line with this idea, large-scale models lump the activity of millions of neurons to emulate realistic brain signals (Freeman, 1977; Nunez and Silberstein, 2000). This modeling approach initiated by Lopes Da Silva et al. (1974) has been widely used to predict the macroscopic electrical activity of the brain. (Freeman, 1978; Stam et al., 1999; Valdes et al., 1999; Wendling et al., 2000; Robinson et al., 2001; David and Friston, 2003; David et al., 2004; Naruse et al., 2010). A large number of these mesoscopic models have been devoted to characterize the alpha rhythms (Lopes Da Silva et al., 1974; Jansen and Rit, 1995; Stam et al., 1999).

Widely studied neural population models are able to generate oscillatory activity (e.g., in the alpha band) through purely cortical connectivity (Wilson and Cowan, 1972) as well as with cortico-thalamic interactions (Lopes Da Silva et al., 1974; Robinson et al., 1997, 2001). More recently, Naruse et al. (2010) proposed a model based on excitatory lateral interactions between coupled cortical macrocolumns serving as alpha generators (Jansen and Rit, 1995). This model was able to reproduce the alpha rhythm, as well as ERPs, and ERS/ERD of the alpha rhythm without involving the thalamus (see Cortico–cortico model in Figure 3).

In a study with EEG resting-state recordings, characteristic non-linear features of the alpha rhythm power were reported (Freyer et al., 2009a). These features are bistability, scale invariance, and dwell time cumulative distributions with the shapes of stretched exponentials. Freyer et al. (2011) were able to reproduce all the above features of empirical alpha oscillations by adding a cortico-thalamic feedback in the extended thalamo-cortical neural field model (see Thalamic-cortico model in Figure 3) (Robinson et al., 1997, 2001). The underlying neural field model incorporated the detailed mathematical description of biophysical factors, such as synaptic and dendritic dynamics, non-linear firing responses, and axonal delays. These temporal features can also be extracted from a simple generic model as shown in Freyer et al. (2012b). The canonical description offers a systematic insight into stochastic and non-linear contributions providing a strong link between empirical data and computational models. Moreover, through model inversion, it is possible to determine the critical point of alpha mode switching in the resting-state. Recently, Lundqvist et al. (2013) used a bistable cortical attractor model to study the effect of prestimulus alpha oscillations on the perception of weak stimuli. In this model alpha oscillations are produced in a default state characterized by low-rate diffuse activity before stimulus onset. Such a state represents a kind of readiness to process stimuli. After stimulation, the network transits other coding states characterized by elevated spiking activity in areas selective to the stimuli. During activation the model produces gamma oscillatory activity in trials with successful detection. Interestingly, the network transitions were modulated by both, the phase and power of the alpha oscillations. The attractor model by Lundqvist et al. (2013) constitutes a plausible theoretical demonstration of the effects that alpha oscillations before stimulus onset have on detection performance.

#### Full-brain models

The spatial structure of resting-state activity investigated in numerous studies predominantly reflects gross anatomical connectivity between brain areas but cannot be understood in those terms alone. Large-scale computational models have studied the relation between anatomical structure and intrinsic node dynamics (Honey et al., 2007; Ghosh et al., 2008; Deco et al., 2009; Freyer et al., 2012b; Ritter et al., 2013). These models often used realistic neuroanatomical information from the macaque brain provided by the CoCoMac data base (Kotter, 2004) and/or from the human provided by diffusion weighted MRI or dwMRI (DTI/ Diffusion Spectrum Imaging) techniques (Hagmann et al., 2008). In particular, Ghosh et al. (2008) and (Deco et al., 2009) considered the full space-time structure of the problem (neuroanatomical connectivity matrix, conduction delays and noise) such that they were able to explain the formation and dissolution of slow fluctuating RSNs by considering very simple local oscillatory dynamics at each node. As an extension, Deco et al. (2009) and Deco and Jirsa (2012) formulated and studied a detailed and realistic spiking attractor network structured in brain areas and connecting these local networks using a neuroanatomical large-scale connectivity matrix obtained from human subjects via dwMRI tractography.

Cognition results from interactions between functionally specialized but spatially distributed brain areas. As multiple brain areas are involved in such computations, full-brain models are necessary to account for the mechanisms leading to cognitive states. In this regard, a newly developed simulation platform, The Virtual Brain (TVB, http://thevirtualbrain.org), provides the necessary tools to perform full-brain simulations (Jirsa et al., 2010; Ritter et al., 2013; Sanz Leon et al., 2013). This neuroinformatics platform simulates full-brain network dynamics taking into account biologically realistic connectivity information. The platform integrates the large-scale structure of brain connectivity; it spans brain regions modeled with descriptions at microscopic and mesoscopic levels (neural networks and neural masses), using realistic local cortical connectivity. Thus, regional dynamics can be evaluated in the context of long-range spatio-temporal interactions and at the same time preserving the perspective on global dynamics of the brain. Models of the microcircuit can finally be put into the functional context, and the large body of theory developed in computational neuroscience on the microscopic scale can be exploited for the investigation of large-scale brain function. Such model-based inferences would establish a strong link across brain scales between the underlying neurophysiological mechanisms and the macroscopic large-scale brain signals observed in different imaging modalities.

## The role of the alpha rythm in perceptual learning

Since ongoing brain oscillations such as the alpha rhythm emerge from the underlying brain architecture (see section *Cellular and Regional Substrates of the Alpha Rhythm*), changes in the structural neural connectivity induced by learning are likely to alter ongoing oscillatory activity. At the same time, plastic mechanisms underlying learning can be boosted during specific brain states defined by spatial patterns of ongoing oscillatory activity (Freyer et al., 2013). While recent studies support this circular relation between learning and ongoing brain activity (e.g., Lewis et al., 2009; Freyer et al., 2012a, 2013), the exact mechanisms behind this interaction are still unclear. Empirical evidence (Freyer et al., 2012a, 2013) shows that perceptual learning is alpha state dependent and that perceptual learning alters locally the coherence of spontaneous alpha activity. However, based on the empirical data alone we cannot answer how local plastic changes alter large-scale brain activity, and how ongoing oscillations affect the neural mechanisms underlying learning. In this section we address these issues. Due to the existing empirical evidence we focus here on the relation between the alpha rhythm and perceptual learning. Perceptual learning can be induced via time-dependent stimulation (Beste and Dinse, 2013), in which case it can be associated to alpha oscillations (Freyer et al., 2012a, 2013). This leads us to believe that the effect of alpha oscillations on perceptual learning is related to time-dependent cellular plasticity. This hypothesis is further detailed in section How Oscillatory Brain States Facilitate Learning. In section How Learning Shapes Ongoing Activity we present evidence showing that learning systematically alters the activity of large-scale functional networks. In addition, we highlight recent computational findings demonstrating that modifications of large-scale functional connectivity may result from plastic alterations in local cortical circuits.

### How oscillatory brain states facilitate learning

Information processing in general and plasticity in particular depend on time-precision that can be provided by periodic signals such as brain rhythms. Neural oscillations are often phase entrained; they can index naturally spike timing and, therefore, they can influence spike-timing dependent plasticity (STPD). In fact, several computational models have successfully linked STDP (see for a review Caporale and Dan, 2008) and brain oscillations (Hosaka et al., 2008; Masquelier et al., 2009; Neymotin et al., 2011). At the same time, large-scale neural oscillations provide spectral fingerprints for neuronal interactions across brain areas underlying visual perceptual learning or other cognitive processes (Engel et al., 2001; Salinas and Sejnowski, 2001; Varela et al., 2001; Siegel et al., 2012). Experiments in animals and humans and computational models have stressed the importance of timing-dependent neural processes for information processing and learning. Extracellular recordings in awake animals have demonstrated that neural spiking activity with respect to the phase of local field potentials (LFP) recorded simultaneously carries complementary information about sensory stimuli (Montemurro et al., 2008; Kayser et al., 2009). In a more recent study, Ng et al. (2013) investigated the relationship between phase of ongoing oscillations and cognitive variables. The authors report that stimulus selective firing patterns imprint on the phase rather than on the amplitude of slow EEG and LFP oscillations. Stimuli that can be discriminated based on firing rates can also be discriminated via oscillatory phase patterns but clearly not via oscillatory amplitude.

**Figure 5:**
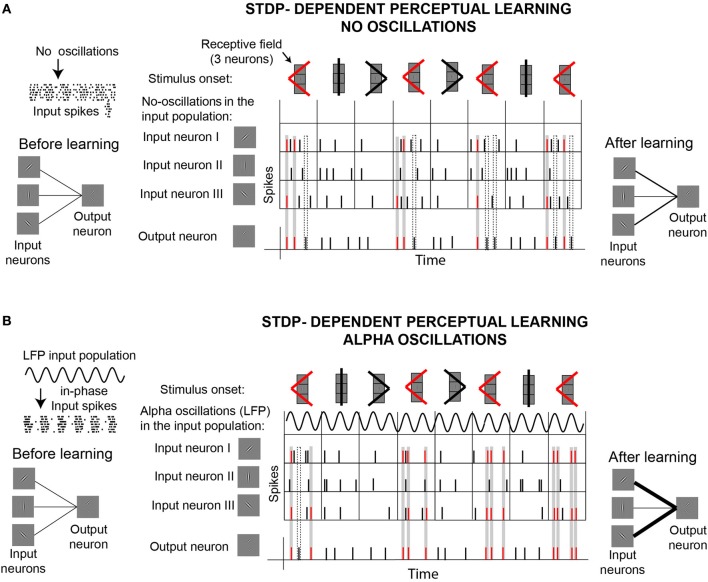
**Illustration of a potential active role of alpha oscillations for perceptual learning, based on recent computational work (Masquelier et al., 2009). (A)** STDP-dependent perceptual learning without background alpha oscillations. Learning is illustrated by the ability of a “shape-selective” neuron (or “output neuron”) equipped with STDP to detect the appearance of a target shape (red <), which appears more frequently than two other shapes. As it is illustrated on the left side of the figure, the output neuron receives input from three “orientation-selective” neurons. The three input neurons are connected to the output neuron sharing the same synaptic strength before the stimulation starts. The top row in the central panel illustrates the presentation of the stimuli (shapes), which stimulate the three input neurons. The three input neurons are arranged in a column, responding in this way to specific regions of the shapes (receptive fields). The second row in the central panel indicates that there are no oscillations (LFP) driving the spiking activity of the input neurons. The following rows show the spiking activity of the three input neurons and the output neuron. Since the output neuron is equipped with spike-timing-dependent-plasticity (STDP), the strength of the synapsis connecting the input neurons to the output neuron can change throughout the stimulation according to standard Hebbian rules. During the course of the stimulation, synaptic weights are reinforced whenever input and output spikes coincide within a certain time window, indicated by the gray rectangles. Coincidence of spikes marked in the gray rectangles result in synaptic reinforcement that facilitates the recognition of the target shape (red <). The dashed rectangles indicate some cases in which the spikes of the output neuron don't lead to synaptic reinforcement. At the end of the stimulation, synaptic weights connecting input neurons I and III to the output neuron are reinforced. This reinforcement improves the detection of the target shape. **(B)** STDP-dependent perceptual learning with background alpha oscillations. Spikes of the input neurons are driven by the stimulus and by ongoing oscillations (alpha LFP), which modulate the membrane potential of the input neurons producing “in-phase” spikes. Output neurons equipped with STDP can learn better to detect coincidences of in-phase spikes, compared to the case where no oscillations are involved. At the end of the stimulation, connections between output neurons and neurons I and II are stronger in the presence of alpha oscillations **(B)** than without **(A)**, as indicated on the right side of the figure. This implies that the output neuron in case **(B)** can detect better the presence of the target shape (red <) than the output neuron in **(A)**.

Concurrent modeling work demonstrates the importance of rhythmic input and STDP on downstream learning (Masquelier et al., 2009). Masquelier et al. (2009) showed how a single downstream neuron equipped with STDP can decode a repetitive input pattern encoded in the oscillatory phases of a subset of afferents (~10%). While a role of STDP in pattern detection is well established (Masquelier et al., 2008), the demonstration that the encoding of patterns in oscillatory phases facilitates learning is novel. An interesting finding of this work is that, while oscillations in the alpha range proved to be good for STDP-learning, oscillations in the gamma range (>40 Hz) turned to be not optimal. Figure 5 illustrates these ideas by showing the behavior of a neuron equipped with STDP that learns to detect a target shape. This “output” neuron receives inputs from three different “orientation-selective” neurons. Depending on their receptive fields, input neurons respond to different parts of the shapes. In one situation (Figure 5A), the only external force driving the responses of the input neurons are the stimuli. In the second situation (Figure 5B), the spikes generated by the input neurons depend on two external forces, the stimuli and an oscillatory signal (LFP) that modulates the membrane potential of the input neurons. In contrast to the former case, in the later case the LFP oscillations influence the spiking probability of the input neurons, which in turn generate “in-phase” input spikes, as illustrated on the left side of the figure. Given the phase information carried by in-phase spikes, neurons capable of STDP can learn better to detect the target shape. The effect of oscillatory information in the inputs of neurons with STDP can be generally applied to achieve different forms of learning. Based on the idea that “phase-of-firing coding” has a major impact on downstream learning and decoding when associated with well established STDP, we hypothesize an “active” role of alpha oscillations in stimuli encoding and memory acquisition. Furthermore, we consider that alpha oscillations could fulfill a similar role modulating the efficacy of perceptual learning, as a particular case of the mechanisms proposed by Masquelier et al. (2009).

**Figure 6:**
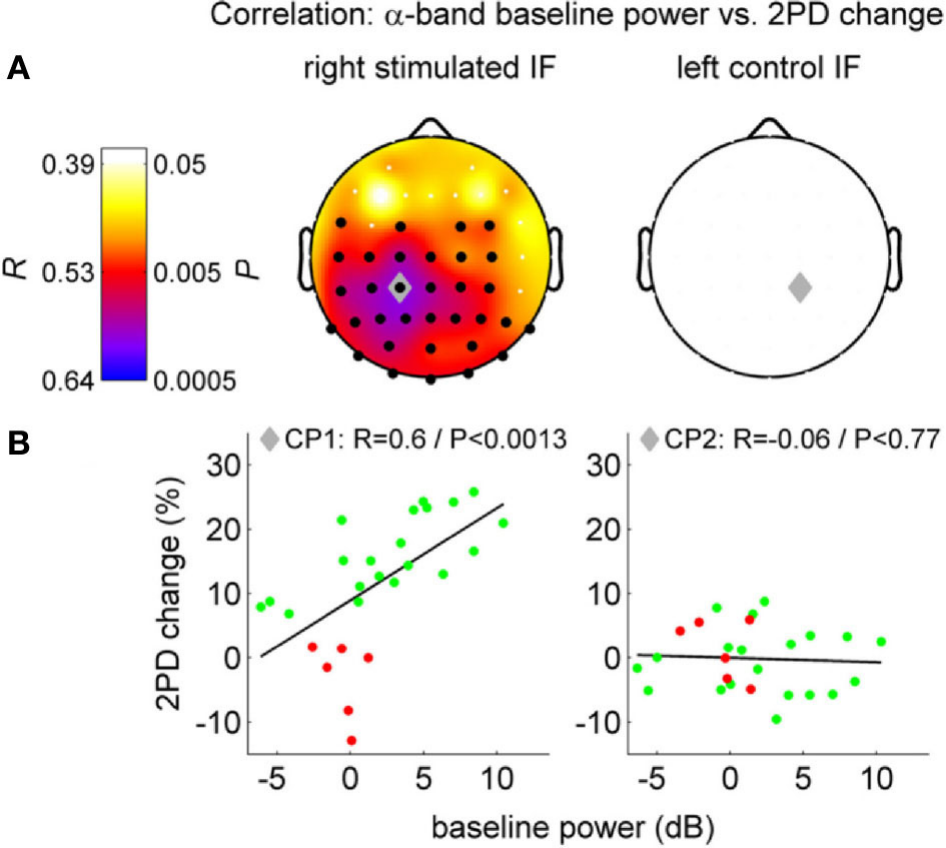
**Correlation between two-point discrimination (2PD) change and alpha band resting-state EEG power before perceptual learning (left column: stimulated side, i.e., right finger; right column: control side). (A)** Top row, scalp distributions with Pearson's correlations coefficients (*R* and *P*-values). Black dots: significant cluster of channels. Gray diamond: maximum correlation. **(B)** Scatter plot, single subject values at channels CP1/CP2 (green: successful learners, red: bad learners). Figure adapted with permission from Freyer et al. (2013).

It is likely that the interaction between alpha oscillations and perceptual learning occurs by means of time-dependent information processing. However, this interaction might occur at different times of the learning process, and involving brain mechanisms that work at different time scales. Consequently, alpha oscillations and perceptual learning may interact differently before, during and after training or stimulation. Analogously, this interaction may differ depending on whether we consider 1 min, 1 h, or 1 day before or after learning. A recent study on a visual perceptual task (Hamame et al., 2011) investigated the relation between perceptual learning and alpha oscillations at different epochs of the learning process. They found an intriguing relationship between alpha and perceptual learning by correlating changes in performance with different oscillatory features during training. They observed a complex modulation of the amplitude of both, the alpha and gamma bands, along the course of training. Another recent study set out to investigate the effect of neural oscillations on perceptual learning before sensory stimulation was applied (Freyer et al., 2013). Freyer et al. (2013) asked to what extent different ongoing neuronal states of individual subjects before repetitive somatosensory stimulation are able to explain individual differences in the learning success (Freyer et al., 2013) (Figure 6). The authors found that ongoing alpha oscillations over sensorimotor areas contralateral to the stimulated side before stimulation positively correlate with the learning outcome induced by the stimulation. The higher the alpha power was before the stimulation, the more the subjects improved their tactile sensitivity after the stimulation. Performance improvement and the correlation with the alpha rhythm was present only in the stimulated finger and the alpha power in the contralateral somatosensory region. Neither the behavioral improvement nor the correlation with alpha was observed in the control condition (in the non-stimulated finger of the other hand). This pattern of results suggests that the effects of alpha in learning cannot be explained by global fluctuations of attention or vigilance. Instead, these finding indicates alpha band specific plastic mechanisms localized in the sensorimotor cortex and other areas implicated. According to the theory “gating-by-inhibition” (Jensen and Mazaheri, 2010), high alpha activity before stimulation in the study by Freyer et al. (2013) could reflect an “idle state” or inhibited state in learning-relevant areas. Such a state can be regarded as a standby mode that allows the system to reorganize rapidly when stimulation starts. Beyond this, considering the “timing role” of alpha oscillations detailed above, prominent alpha activity before stimulation could contribute to generate the necessary oscillatory background that combined with STDP facilitates learning in sensorimotor areas once the stimulation starts.

### How learning shapes ongoing activity

Under resting-state conditions –in the absence of a particular task or stimulation– the brain yields networks of coherently fluctuating ongoing activity that delineate various well-known functional neural networks (Fox and Raichle, 2007; Smith et al., 2009). While resting-state brain activity predicts behavioral performance in various tasks (Hampson et al., 2006; Fox et al., 2007; Mennes et al., 2011; Zou et al., 2013), sensory experience and learning leave traces in the ongoing brain activity (Harmelech and Malach, 2013). For example, changes in RSNs have been demonstrated following semantic-matching (Wang et al., 2012) and classification (Stevens et al., 2010), short- and long-term motor learning (Albert et al., 2009; Ma et al., 2011; Taubert et al., 2011; Vahdat et al., 2011), or associative encoding (Tambini et al., 2010). Experiments on visuomotor coordination have shown that learning lead to plastic changes that can be observed in electrical oscillations recorded with EEG during the resting-state and during sleep. Following learning in a visuomotor task, investigators observed an increase in sleep slow wave activity (EEG power density between 0.5 and 4.5 Hz) in the right parietal area (Huber et al., 2004; Maatta et al., 2010; Murphy et al., 2011). During wakefulness in contrast, an alpha decrease over the same region and an increase over left parietal and right frontal areas has been reported (Landsness et al., 2011). The authors report correlations between the behavioral outcome of learning and the EEG signatures during wakefulness and sleep. This results evidence the impact of learning on large scale functional networks in visuomotor coordination (Landsness et al., 2011). Taken together, as Harmelech and Malach (2013) hypothesize in their recent review, spontaneous brain activity not only reflects “external statistical structures” but they also seems to reflect “the entire set of individual inner cortical and cognitive biases” which partially depend on past experiences (learning).

In this line, several studies show that perceptual learning affects large-scale resting-state BOLD signals (Lewis et al., 2009; Baldassarre et al., 2012; Ventura-Campos et al., 2013). Using a shape identification task, Lewis et al. (2009) show learning-related modulations in resting-state BOLD functional connectivity. They demonstrate that visual perceptual learning can modify networks that are recruited during the course of training. Lewis et al. (2009) report increased resting-state fMRI functional connectivity between parietal and visual cortex after visual perceptual learning. Additionally, after perceptual learning the visual cortex and fronto-parietal attention areas were negatively correlated. The higher the (negative) correlation was, the more subjects improved their performance. Ventura-Campos et al. (2013) combined task-related and resting-state fMRI to investigate their relation to phonetic learning. The authors showed that resting-state functional connectivity between the left insula/frontal operculum and the left superior parietal lobe measured before training predicts individual learning outcomes. Using EEG measurements, a recent study revealed altered functional connectivity after 30 min of HFS –capable of inducing perceptual improvement (Pleger et al., 2001; Freyer et al., 2013)—as indicated by an increase of local resting-state alpha coherence within distributed sensorimotor cortical areas, contralateral to the stimulated side (Freyer et al., 2012a). Despite the sensory stimulation is applied to the of tip a single finger, in the study by Freyer et al. (2012a) clear large-scale effects on oscillatory alpha activity could be observed over distributed sensorimotor regions.

To understand the interaction between long-range brain communication and local synaptic processes, full-brain models such as the TVB are suitable simulation frameworks (see section Full-Brain Models). Currently, such models are able to reproduce coarse/general aspects of spatiotemporal brain dynamics that are present in the majority of experimental data. In these models, brain activity can be constrained by individual large-scale connectivity parameters derived from dwMRI. This allows scientists to simulate the effects that large-scale plastic changes have on emerging brain dynamics. Our hypothesis of how plastic changes in sensory areas elicited by perceptual learning affect large-scale information processing is based on a recent study of our group, that shows results of full-brain simulations (Roy et al., in preparation). In this study, Roy et al. (in preparation) propose ways to incorporate plasticity mechanisms in existing computational models that are capable of generating ongoing spontaneous activity as a function of transmission delays, noise and connectivity. In those simulations, plasticity in local populations can change the dynamical stability of global functional networks distributed across multiple brain areas. The main findings are: (1) Local network activity in the absence of plasticity is characterized by irregular oscillations between a low-amplitude asynchronous and a high amplitude synchronous state. (2) Alterations in local synapses (due to STDP), in the order of few milliseconds, induce changes in the local connectivity of the brain areas where plasticity is implemented. Such changes alters distinct features of the global functional connectivity (FC). (3) The interaction between those regions is organized systematically in correlated and anti-correlated networks depending on the choice of the model parameters. These parameters include plasticity parameters as well as the amplitude, frequency of the background oscillatory state. Anti-correlated networks after time dependent plasticity show significantly and highly correlated BOLD spatiotemporal activity. In particular, simulations show that the intrinsic alpha oscillations generated by local cortical neurons efficiently influence the learning outcome of brain areas connected structurally. While this model does not target exclusively brain areas involved in perceptual learning, it proposes a general mechanism able to explain the effect of perceptual learning on resting-state activity, as it is the case in some of the studies presented above. In the near future, based on individual subject/patient parameters, this kind of model simulations will help to predict the impact that interventional actions, which evoke plasticity will have on evolving brain dynamics. Ultimately, similar brain simulations will aid scientists in the planning of experimental learning protocols as well as clinicians developing therapeutic strategies, in order to reveal the complex relation between perceptual learning and large-scale ongoing brain activity.

## Conclusions and outlook

The current view on how alpha oscillations relate to cognitive abilities, such as perceptual learning, is becoming far more complex compared to the initial view which associates alpha activity to an “idle” brain state. Although the role of alpha oscillations in spatial attention, working memory, and perception is well documented, and despite initial evidence indicating that alpha oscillations influence perceptual learning, the detailed role of alpha rhythm in perceptual learning and its contribution to the observed variability in learning outcome needs further empirical and theoretical assessment. Changes in perception due to perceptual learning develop under a variety of conditions such as training, sensory or even central stimulation, with and without attention, showing complex degrees of spatial specificity and temporal persistence. This suggests that a variety of neural processes and brain areas are implicated in perceptual learning (Sasaki et al., 2010) that may be subject to interaction with alpha oscillations. Alpha oscillations prior to and after learning may increase the gain of neurons through a dynamic balance of excitation and inhibition (Haider et al., 2006; Raichle, 2006). Increasing the gain of neurons has an effect on their responsiveness to input which is critically important for perception and attention (Salinas and Thier, 2000)—both processes that interact with perceptual learning. Another potential interaction mechanism may be time-dependent plasticity. Phase information could serve as an internal reference for spike trains representing input signals, improving input processing, and memory consolidation (Masquelier et al., 2009). The combination of different measures including behavior, brain activity with high temporal and spatial resolution, as well as computational modeling will be crucial to overcome the difficulties of understanding the precise link between ongoing oscillations such as alpha activity and perceptual learning, and for utilizing those insights in the clinical and real-life setting.

### Conflict of interest statement

The authors declare that the research was conducted in the absence of any commercial or financial relationships that could be construed as a potential conflict of interest.
